# From Academic Involution to Psychological Distress: The Mediating Role of Competitive Psychology in Depression–Anxiety–Stress Among College Students in Anhui Province

**DOI:** 10.3390/bs16071201

**Published:** 2026-07-16

**Authors:** Xiaoyan Qi, Fang Cheng, Min Wang

**Affiliations:** 1 School of Nursing, Anhui Medical University, Hefei 231200, China; 2022500009@ahmu.edu.cn; 2 The Second People’s Hospital of Hefei, Hefei 230011, China; 3 Huangshan City People’s Hospital, Huangshan 245000, China

**Keywords:** competitive psychology, involution, depression–anxiety–stress, social comparison theory, conservation of resources theory, cross-sectional design

## Abstract

**Aim**: This study aims to explore how competitive psychology mediates the relationship between involution and depression–anxiety–stress among college students from three universities in Anhui Province, China. **Background**: Against the global involution trend, college students excessively pursue academic achievements, leading to heightened self-involution and psychological issues like anxiety and depression, while competitive psychology’s mediating role here remains under-explored. **Methods**: A multi-cross-sectional survey in three Anhui universities used convenience sampling to collect 592 samples. **Results**: Three validated scales assessed involution, competitive psychology, and depression–anxiety–stress, with mediation analysis via SPSS Process 3.3. The results showed strong positive correlations between the three variables (r = 0.827, 0.638, *p* < 0.001), and competitive psychology significantly mediated the involution–depression–anxiety–stress relationship (r = 0.110). **Conclusions**: The observed data are consistent with a correlational mediation model among college students from three universities in Anhui Province. The findings provide theoretical references for local university educational practice and mental health guidance, without causal inference or nationwide policy generalization; future research should explore related mechanisms and interventions. **Clinical Relevance**: This study provides empirical evidence for nursing and mental health professionals to design targeted psychological interventions for college students, helping mitigate the negative impacts of involution and competitive pressure on mental health in clinical practice.

## 1. Introduction

In recent years, the expansion of university enrollment has been regarded by the government as an important measure to promote economic growth and social development. Since the curtain was first raised on the expansion of university enrollment in 1999 (Z. Y. Zhang et al., 2023), according to statistics from the Ministry of Education, by 2022, the number of college graduates is expected to break through the ten million mark for the first time, reaching an estimated 10.76 million people, an increase of 1.67 million compared to the previous year, setting a new historical record (Ministry of Education of the People’s Republic of China, 2023). However, with the influx of a large number of college graduates into the market, the employment market has become highly competitive. A data survey conducted abroad on the employment situation of college students found a clear relationship between their employment status after graduation and their performance during school (Elmansouri et al., 2023). This indicates that better academic performance in college is associated with a higher likelihood of obtaining a satisfactory job after graduation. As a result, college students are compelled to continue engaging in intense academic competition to achieve better grades and future prospects. This has led to a more intensified involution in college students’ learning. The term “involution” originally comes from sociology and economics and has since spread to the field of education, where it is widely used to describe excessive competition in areas such as education and the workplace. Particularly in the field of higher education, with the increasing number of college students, the employment pressure faced by graduates has grown significantly. Many students, in an effort to enhance their competitiveness, have no choice but to participate in various activities such as obtaining certificates, preparing for postgraduate entrance exams, and internships. This creates a state of intense competition that appears diligent but is, in reality, limited in effectiveness, and this phenomenon is referred to as “involution.” While moderate involution can stimulate students’ internal motivation and encourage them to study harder, in the long run, a sustained high-intensity involution learning environment not only fails to benefit personal growth but also poses certain risks (Yi et al., 2022). On one hand, in the continuous and seemingly purposeless learning involution, studying becomes a habitual pseudo-learning operation, losing the inherent meaning and pleasure of learning. On the other hand, relevant research has found that an involutionary academic atmosphere can be correlated with students’ academic involution and a sense of relative deprivation, ultimately resulting in stress and a range of psychological problems (A. Liu et al., 2024). Anxiety, depression, and stress are common manifestations of psychological issues. Although they may manifest at different levels, anxiety, depression, and stress often coexist in individuals, especially among student groups facing such high academic loads (Shamsuddin et al., 2013). A study by Chan et al. (2023) found that when the human body is under high stress, it releases stress signals that are transmitted from the central nervous system to immune cells to regulate stress-related behaviors. High involution has permeated all aspects of people’s lives and is typically manifested in the maintenance of personal competitiveness. A typical example is in highly competitive environments, where individuals continuously increase their efforts and investments to maintain their competitiveness. However, this increased effort is not associated with corresponding gains but may instead be associated with greater work and life pressures. This phenomenon is particularly pronounced in China, with its large population (Y. Liu et al., 2022). Living long-term in a highly involuted environment, competitive pressure can be correlated with individuals feeling a greater psychological burden. Individuals may sacrifice personal time and health to cope with competition, which is correlated with an imbalance between work and life, which may have a negative impact on mental health as they constantly strive to outdo others to maintain their competitiveness (Dou et al., 2022). This study aims to explore the correlation of college student involution on individual anxiety, depression, and stress through a competitive mindset. Specifically, we propose that in an involutionary environment, college students tend to compare their performance and abilities with those of their peers to assess their social and capability status. This comparison may be associated with intense competitive psychology. This hypothesis is based on the theoretical foundation of social comparison theory (Festinger, 1954) and conservation of resources theory (COR, Hobfoll, 1989). Social comparison theory posits that individuals possess an intrinsic drive to evaluate their own opinions and abilities, tending to determine their social and competence status through comparison with others. In the context of involution, college students are compelled to engage in frequent upward social comparisons. Such comparisons not only fail to produce self-enhancement motivational effects but instead trigger threatening self-evaluations, thereby activating competitive psychology. Meanwhile, conservation of resources theory emphasizes that individuals have an innate tendency to protect existing resources and acquire new ones. When perceiving resource loss or investments that fail to yield corresponding returns, significant psychological distress ensues. The essence of the involution phenomenon is precisely “effort inflation”—individuals invest substantial resources (time, energy, health) yet fail to obtain expected returns (better grades, employment opportunities). This state of resource depletion and benefit imbalance directly threatens individuals’ psychological resource equilibrium. On this basis, we further incorporate self-determination theory (Deci & Ryan, 2000) to reveal the correlational pathway through which competitive psychology transforms into psychological distress. This theory maintains that the satisfaction of autonomy, competence, and relatedness needs constitutes the foundation of mental health. However, competitive psychology in an involutionary environment is often accompanied by enhanced external control (compelled participation in competition) and weakened intrinsic motivation (learning shifts from interest-driven to fear-driven), leading to frustration of basic psychological needs and ultimately manifesting as symptoms of anxiety, depression, and stress. This integrated theoretical framework not only explains “why involution affects mental health” but also clarifies “why competitive psychology serves as the critical transformation mechanism”—it acts as a bridging variable between social comparison triggers and psychological resource depletion. These three theories are not simply juxtaposed, but constitute a multi-level, dynamic explanatory chain: social comparison theory explains how the involutionary environment is perceived (situation→cognition); conservation of resources theory explains why competitive psychology generates stress (cognition→resource threat); and self-determination theory explains how stress transforms into symptoms (resource threat→need frustration→psychological distress).

Previous studies have indirectly investigated the relationship between involution, competitive psychology, and anxiety and depression (Gilbert et al., 2009). However, there is a lack of research on how competitive psychology acts as a mediator in the relationship between involution and anxiety, depression, and stress, especially among college students. This study will provide scientific evidence for the existing literature by exploring the mediating role of competitive psychology in the relationship between involution and anxiety, depression, and stress. It will focus on a subset of college students in Anhui Province, China. By examining the mediating pathway, this study will deepen our understanding of the mechanisms underlying these relationships and offer more suggestions for the reform and development of the education sector and teaching models.

### 1.1. Literature Review and Hypothesis Development

#### 1.1.1. Involution and Anxiety, Depression, and Stress

In contemporary Chinese society, the phenomenon of involution refers to the excessive competition that occurs when resources are limited, as people vie for these resources, resulting in a decrease in the “return on effort” for individuals, which can be seen as an “inflation” of effort. The phenomenon of involution is reflected in various fields such as education and the workplace. For example, in the field of education, students continuously increase their study time and intensity in order to achieve better grades and opportunities, but the overall gains do not significantly increase (Yan et al., 2022). The emergence of involution is associated with multiple factors, including the limited nature of resources, intensified social competition, and individuals’ pursuit of success. It is not only a social phenomenon but also reflects a social mentality, that is, in a rapidly developing and fiercely competitive social environment, people’s anxiety and pressure regarding personal development and success (L. Liu et al., 2024). Previous research (J. Zhang et al., 2024) found in a study exploring the impact of social support on stress and depression among medical students in the face of an involutionary environment that most students have stress and depression issues. Since stress, depression, and anxiety are interrelated psychological states with complex interactions among them (Chen et al., 2024), we propose the following hypothesis:

**H1.**  *Involution is positively correlated with anxiety, depression, and stress.*

#### 1.1.2. Involution and Competitive Mindset

A study by W. S. Wang et al. (2024), based on the theory of psychological compensation, analyzed different dimensions of competitive pressure, including competitive outcome pressure and competitive process pressure, and how they affect individuals’ psychological compensation strategies, thereby leading to involution or lying flat. It was found that when competitive outcome pressure threatens an individual’s self-esteem and prompts them to adopt a fluid compensation strategy, an involution effect is formed. In a mixed study exploring employees in the Chinese market (Dou et al., 2022), it was discovered that employee involution is a multi-dimensional and richly connotative structure, including four dimensions: inefficient busyness, innovation exhaustion, promotion anxiety, and internal competition. Reducing internal competition among employees requires management to strengthen interventions in employees’ competitive psychology. Amid the deteriorating national employment environment, new-generation employees face higher job demands, and the “involution” culture has become the mainstream culture in various industries. “Involution” has led to the increasing prevalence of the competitive psychological behavior of overtime work among new-generation employees (Niu & Yang, 2022). Therefore, we propose the following hypothesis:

**H2.**  *Involution is positively correlated with competitive psychology.*

#### 1.1.3. Competitive Mindset and Anxiety, Depression, and Stress

According to the principles of social comparison theory, the theory states that individuals evaluate themselves by comparing themselves with others, which may be associated with competitive behavior and perceived stress, thereby affecting individuals’ anxiety and depression emotions (W. Zhang et al., 2024). Many previous studies have identified competitive psychology as a key factor leading to anxiety, depression, and stress (X. Li, 2023; X. S. Liu, 2020). These studies found that when individuals are in a stressful environment and face the pressure generated by a competitive environment, it affects the level of psychological flexibility, thereby indirectly predicting the degree of educational anxiety and depression. In addition, a study conducted by Rabby and Islam (Rabby et al., 2023) showed that in the face of immense academic pressure and unhealthy competition, individuals who are overly competitive may become more irritable and impatient and may have more health problems. Moreover, overly competitive individuals often exhibit high levels of narcissism, strong self-esteem, and higher rates of anxiety and depression. These individuals may need to constantly engage in social activities to gain affirmation and will never be satisfied with themselves unless they achieve their goals. The sources of competitive pressure are diverse, and these sources of pressure can be associated with significant competitive pressure among young people, which may in turn trigger anxiety and depression. Therefore, we propose the following hypothesis:

**H3.**  *Competitive psychology has a positive and significant correlation with college students’ anxiety, depression, and stress.*

#### 1.1.4. The Mediating Role of Competitive Mindset

The mediating role of competitive mindset refers to the mediating variables of an individual’s internal psychological state and behavioral response when facing a competitive situation, which affect anxiety and depression emotions. It is the bridge that transforms the competitive pressure felt by an individual into specific behaviors and emotional states. Early research findings indicated that the strength dimension of the psychological resilience scale, the self-kindness and mindfulness dimensions of the self-compassion scale, and the forgiveness of others and self-forgiveness dimensions of the forgiveness scale are all protective factors for depressive symptoms among college students, while the resilience dimension is a risk factor. This indicates that an individual’s psychological quality plays a certain buffering role when facing competitive pressure (Mu et al., 2019). Social comparison theory states that individuals evaluate themselves by comparing themselves with others, which may be associated with competitive behavior and perceived stress, thereby affecting individuals’ anxiety and depression emotions. In addition, conservation of resources theory also mentions that competition may trigger an individual’s resource conservation behavior to reduce anxiety and stress (Wen et al., 2022).

Moreover, the relationship between the social comparison effect and involution tendency, in a study based on the mediation model of competence need satisfaction, found that the social comparison effect influences involution tendency through the sense of competence need satisfaction, which plays a mediating role in involution psychology (M. Zhang et al., 2023). The sense of competence need satisfaction is usually associated with an individual’s intrinsic motivation and self-esteem. A competitive mindset can to some extent reflect and shape an individual’s self-esteem. In self-determination cognitive motivation theory, individuals need to balance success and failure in competition, as well as intrinsic and extrinsic motivation, to maintain a healthy sense of self-esteem and a positive competitive attitude (Bao & Zhang, 2005). Therefore, we propose the following hypothesis:

**H4.**  *Competitive psychology plays a mediating role between involution and anxiety, depression, and stress.*

**Figure 1:**
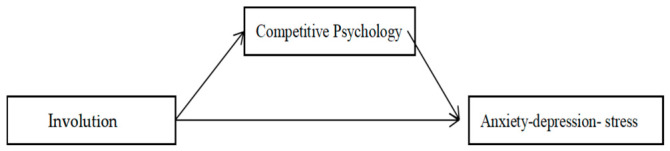
Conceptual frame developed for this study.

Based on the previous literature review and hypothetical questions, the conceptual framework of this study is shown in Figure 1.

This study further clarifies the connotation of academic involution specific to Chinese college students, and constructs a multi-theory mediated framework that distinguishes behavioral involution from individual competitive psychology. Unlike previous studies that mainly focused on simple correlations among stress, anxiety and competition, the present study further identifies competitive psychology as a proximal mediational mechanism, rather than a simple parallel influencing factor. This provides an incremental theoretical contribution by clarifying the psychological pathway from the involutionary environment to psychological distress, and offers targeted empirical evidence for understanding college students’ mental health under the unique contextual background of academic involution in China.

## 2. Methods

### 2.1. Data Collection

We eventually distributed 620 questionnaires via Wjx.cn (https://www.wjx.cn, accessed on 10 October 2024), a Chinese online data collection website). We used an anonymous online questionnaire that was filled out through WeChat or email. We contacted the relevant teachers at the universities for training before the data collection. The questionnaires were distributed by counselors from three different universities in China through group links. The sampling of college students from each university was conducted successively. All participants included in the study filled out the questionnaire online by scanning a QR code with WeChat, and they were required to complete it within 30 min. No trained personnel were present during the filling process to ensure the authenticity of the responses. A total of 613 college students initially completed the survey. During data cleaning, 6 questionnaires with identical repetitive responses and 15 unqualified questionnaires (including extremely short completion time, regular random filling, and contradictory answers) were further excluded, leaving 592 valid questionnaires for final analysis. The effective response rate was 99%. Sample size justification was based on conventional mediation research standards. A minimum of 500 participants is recommended for stable mediation model testing, and the final valid sample of 592 exceeded the required threshold, providing adequate statistical power for correlation, regression and bootstrap mediation analyses. This study adopted a cross-sectional survey design for the following reasons: Firstly, as involution is a pervasive phenomenon in current Chinese universities, its effects possess immediacy and persistence; cross-sectional data can capture this stable psychological state. Secondly, the core objective of this study was to establish association patterns among variables and test theoretically predicted mediation pathways, rather than to establish causal sequences. We fully recognize the limitations of cross-sectional design in mediation analysis: common method bias may inflate associations among variables, and reverse causality cannot be ruled out (e.g., mental health status influencing individuals’ perception of the involutionary environment and competitive responses).

To address these limitations, we adopted the following measures: (1) Employed Harman’s single-factor test and latent variable control methods to assess common method bias; (2) explicitly established the temporal logical sequence of variables in theoretical derivation; and (3) avoided causal inferences in result interpretation, using expressions such as “predictive effects” and “associational pathways.” The results of this study should be understood as association patterns consistent with theoretical predictions, providing hypothesis foundations and effect size references for subsequent longitudinal research.

### 2.2. Measures

We collected data using an online questionnaire, which mainly included a general demographic questionnaire and the College Student Competitive Psychology Scale. The demographic questionnaire included basic information such as gender, ethnicity, only child status, holding a class officer position, family atmosphere, romantic relationship status, interpersonal relationships, age, weekly extracurricular study time, weekly exercise frequency, daily mobile phone usage time, and competitive psychology. The College Student Competitive Psychology Scale (CPS-CS) was developed by Cen (2009) based on this theory. The scale consists of 23 items and includes five dimensions: excessive competitive attitude, personal development competitive attitude, competitive emotions and feelings, competitive motivation, and competitive interpersonal relationships. The total score and subscale scores of the CPS-CS are all rated on a 5-point scale, with higher scores indicating stronger competitive psychology. The Cronbach α for the CPS-CS is 0.718. The Cronbach α coefficients for excessive competitive attitude, competitive motivation, personal development competitive attitude, and competitive interpersonal relationships are 0.654, 0.655, 0.776, and 0.532, respectively. The omega reliability for the CPS-CS is 0.539, Considering that this study only adopted the total score of competitive psychology for correlation, regression and mediating effect analysis, rather than separate subscale analyses, the relatively acceptable reliability of the total scale can fully support subsequent statistical inference. The slightly lower internal consistency of individual subscales may be attributed to regional sample characteristics and the localized adaptation of the scale among Anhui college students, which is still within the tolerable range for exploratory psychological research. Given the stable structural validity of the original scale reported in the literature and the adequate total-scale reliability, we retained the full scale and only utilized its total score in the current modeling. with subscales ranging from 0.532 to 0.780. This study selected competitive psychology as the mediating variable. The present study deliberately chose competitive psychology rather than other commonly adopted mediators such as social comparison, relative deprivation, academic burnout, perceived stress, need frustration, self-efficacy, and coping style.

The main reasons lie in theoretical connotation and structural pathway matching: social comparison serves more as an antecedent situational cognition rather than a proximal mediating psychological state; relative deprivation, academic burnout and perceived stress mainly reflect passive emotional outcomes after pressure accumulation; self-efficacy and need frustration are broad psychological traits without specific binding to the nature of academic involution; and coping style focuses on post-stress behavioral adjustment rather than the cognitive formation process of psychological distress. By contrast, competitive psychology closely corresponds to the core feature of involution as excessive and ineffective internal competition, exactly occupies the cognitive appraisal link between involution environment and mental health outcomes, and fits well with the multi-theory framework of this study. According to social comparison theory, the direct psychological consequence of upward social comparison is the activation of competitive consciousness, rather than immediate emotional distress. Competitive psychology represents the core content of the cognitive appraisal stage, that is, individuals evaluate the involutionary environment as a competitive arena requiring contention for limited resources. Secondly, compared to other potential mediating variables (such as self-efficacy, psychological resilience, and social support), competitive psychology possesses conceptual tightness with the involution phenomenon: the essence of involution is ineffective competition, and competitive psychology is precisely the direct psychological manifestation of this competition at the individual level. Although variables such as self-efficacy may also mediate the relationship between involution and mental health, they more reflect individuals’ general cognitive evaluations rather than specific reactions to competitive situations. Finally, from an intervention practice perspective, competitive psychology possesses operability and modifiability, providing a clear intervention target for college mental health education.

The College Student Involution Behavior Scale was developed by Yi et al. (2022) based on the theory of internalized involvement behavior to assess involution behavior among college students in Anhui Province. The questionnaire consists of 26 items and includes three dimensions: Factor 1 includes 8 items related to achievement motivation involution, Factor 2 includes 5 items related to reward-oriented involution, and Factor 3 includes 13 items related to passive involution. The scale uses a 5-point Likert scale ranging from 1 = “strongly disagree” to 5 = “strongly agree,” with a total score ranging from 26 to 130. Higher scores indicate greater involvement in involution behavior. The KMO coefficient is 0.936, and the Bartlett’s test of sphericity is significant (*p* < 0.001). The reliability of the scale is α = 0.925. The three dimensions are moderately correlated with each other (r = 0.26–0.69, *p* < 0.01) and highly correlated with the entire scale (r = 0.67–0.87, *p* < 0.01), indicating good validation. The model of the scale is suitable for factor analysis, with acceptable fixed indices and high factor loadings confirming the good structure of the scale. The items are randomly dispersed, effectively avoiding potential biases in the questionnaire collection process. The Depression–Anxiety–Stress Scale (DASS-21) consists of 21 items and includes three dimensions: depression, anxiety, and stress. Items are rated on a 4-point scale ranging from 0 (never) to 3 (always). Higher scores indicate more severe symptoms. For depression, scores ≤ 9 are considered normal, 10–13 mild, 14–20 moderate, 21–27 severe, and ≥28 very severe. For anxiety, scores ≤ 7 are normal, 8–9 mild, 10–14 moderate, 15–19 severe, and ≥20 very severe. For stress, scores ≤ 14 are normal, 15–18 mild, 19–25 moderate, 26–33 severe, and ≥34 very severe. The Cronbach α for the depression, anxiety, and stress subscales are 0.77, 0.79, and 0.76, respectively, with a total-scale Cronbach α of 0.89. The construct reliability for the three subscales is 0.72, 0.80, and 0.76, respectively. Depression–anxiety–stress (DAS) was selected as the outcome variable because its three-dimensional structure can comprehensively capture the emotional response patterns in an involutionary environment. Involution not only leads to singular depression or anxiety but generates a persistent, diffuse psychological stress state. From a clinical relevance perspective, DAS symptoms are the most common presenting complaints in college counseling centers; selecting this outcome variable facilitates the practical translation of research findings. In this study, raw DASS-21 subscale scores were not multiplied by two; severity classifications were interpreted directly based on original raw scores.

### 2.3. Transparency and Openness Statement

This study fully reports the sampling plan, all data exclusion rules (if any), variable manipulations, and all measures, and we adhered to the Journal of Applied Psychology methodological checklist. Analysis code and research materials have been prepared and will be uploaded to a publicly accessible, stable repository (e.g., OSF, Zenodo, or an institutional data repository) upon manuscript acceptance, complete with a DOI and persistent link. De-identified participant data will be shared in accordance with ethical approval requirements. Data were analyzed using SPSS 24.0 and the PROCESS 3.3 macro. The study design and analysis plan were not preregistered.

### 2.4. Statistical Methods

Data were analyzed using SPSS 24.0 software. For normally distributed continuous data, results are presented as mean ± standard deviation. Normality distribution of continuous scale scores was checked via skewness and kurtosis; all variables approximated normal distribution and satisfied the assumptions of linear regression. Residual diagnostics including independence, homoscedasticity and approximate normality of residuals were examined; no obvious violation of linear regression assumptions was observed. Pearson correlation analysis was used to assess the correlation between variables, and linear regression analysis was employed to examine the dependency relationships. Mediation analysis was conducted using the Process 3.3 plugin in SPSS software following Hayes’ (2022) analytical framework. The significance test used the bootstrap method, with Model 4 selected, 5000 resamples, and 95% bias-corrected and accelerated (BCa) confidence intervals adopted for mediation effect evaluation. The significance level was set at α = 0.05. Given multiple statistical tests were conducted, a conventional strict significance level of α = 0.05 was adopted, and results were interpreted cautiously to avoid Type I error inflation. Categorical covariates were dummy coded prior to regression analysis: gender (1 = female, 0 = male), ethnicity (1 = minority, 0 = Han), only child (1 = yes, 0 = no), class cadre (1 = yes, 0 = no), family atmosphere (1 = average/poor, 0 = harmonious), romantic relationship (1 = single, 0 = in relationship), and interpersonal relationship (1 = average/poor, 0 = good). In this study, H1–H4 were predefined confirmatory hypotheses based on theoretical frameworks and the existing literature, while additional post hoc correlation comparisons were regarded as exploratory analyses. To control potential Type I error inflation, the interpretation of *p*-values was treated cautiously, and the main conclusions were based on effect size, bootstrap 95% CIs for total, direct and indirect mediation effects, and theoretical consistency rather than merely relying on statistical significance. Consistent with mainstream reporting conventions for bootstrap mediation analysis, we collectively reported the 95% confidence intervals of mediation pathway effects in the corresponding tables. No multiple comparison correction was applied, but all exploratory findings were interpreted tentatively.

## 3. Results

### 3.1. General Information of the Survey Participants

Among the 592 students, there were 283 males and 309 females; their ages ranged from 17 to 32 years (mean age 20.94 ± 2.43 years). The ethnic distribution included 509 Han Chinese and 83 from other ethnic minorities. There were 398 non-only children and 194 only children. A total of 224 students held positions as class or student union officials, while 368 did not. A total of 339 students reported a harmonious family atmosphere, whereas 253 described their family atmosphere as average or poor. A total of 214 students were in a romantic relationship, and 378 were single. A total of 287 students reported good interpersonal relationships, while 305 had average or poor interpersonal relationships. On average, the 592 students spent 2.02 ± 0.97 h per day on extracurricular study, exercised 2.81 ± 1.14 times per week, and used their mobile phones for 2.69 ± 0.89 h per day (Table 1).

### 3.2. Common Method Bias Test

The Harman single-factor test was used to analyze the common method bias for all items of the involution scale, the competitive psychology scale, and the depression–anxiety–stress scale. The results showed that there was a total of five factors with eigenvalues greater than 1. The variance explained by the largest common factor was 21.917%, which is below the reference value of 40%. Therefore, this study does not have a serious common method bias problem. It should be acknowledged that Harman’s single-factor test is relatively conservative and cannot completely rule out potential common method bias, as this approach has been recognized as a limited diagnostic method in methodological research. To reduce common method variance procedurally, this study adopted anonymous questionnaire submission, randomized item order, no forced missing responses, and a restricted completion time during data collection. Strict procedural controls were implemented to minimize subjective response bias. Given the cross-sectional research design and sample characteristics, further sophisticated methods such as common latent factor adjustment and CFA-based model comparison were not conducted in the current study, which should be recognized as a methodological limitation.

### 3.3. Analysis of the Scores of Involution, Competitive Psychology, and Depression–Anxiety–Stress in the Study Subjects

The total score on the involution scale for the 592 study subjects was 73.98 ± 26.13, the total score on the competitive psychology scale was 65.60 ± 19.67, and the total score on the depression–anxiety–stress scale was 44.78 ± 18.34. The detailed scoring for each dimension of the three scales is shown in Table 2.

The total involution score was positively correlated with the total competitive psychology score and the total depression–anxiety–stress score among the 592 study subjects (r = 0.827, 0.638, *p* < 0.001), as detailed in Table 3. Multicollinearity and construct distinguishability between involution and competitive psychology were further examined. The variance inflation factor (VIF) value between the two variables was below 3.0, indicating no serious multicollinearity. Although the two constructs showed a strong correlation (r = 0.827), they possess clear theoretical and operational boundaries. Academic involution reflects external behavioral characteristics of inefficient academic investment and homogeneous competitive involvement, whereas competitive psychology captures individuals’ internal cognitive tendencies, competitive motivation and emotional orientations toward competition.

The two constructs were measured by two independently developed and validated scales with different item pools and dimensional structures, which further confirms that they are not conceptually identical. The high correlation only reflects the inherent linkage between competitive behaviors and corresponding psychological states.

Based on the above evidence of non-collinearity and construct differentiation, the two variables were retained for subsequent mediating model analysis.

### 3.4. Mediating Effect of Competitive Psychology on the Relationship Between Involution and Depression–Anxiety–Stress in the Study Subjects

**Figure 2:**
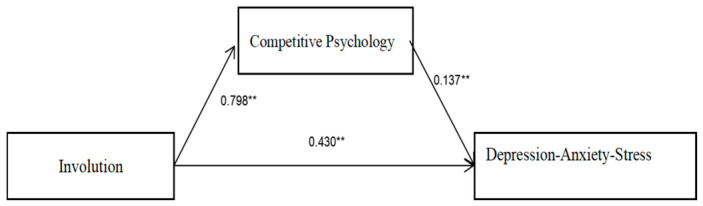
Mediating effect model of competitive psychology between involution and depression–anxiety–stress in the study subjects. Note: ** represents significance levels of *p* < 0.01.

After standardizing the total involution score, total competitive psychology score, and total depression–anxiety–stress score, linear regression analyses were first conducted separately with competitive psychology and depression–anxiety–stress as dependent variables and involution as the independent variable. Then, a regression analysis was performed with depression–anxiety–stress as the dependent variable and both involution and competitive psychology as independent variables. In each regression analysis, gender, age, ethnicity, only child status, class or student union officer status, family atmosphere, romantic relationship status, interpersonal relationships, average daily extracurricular study time in the past week, average weekly exercise frequency, and average daily mobile phone usage time were included as covariates. All three regression models were statistically significant, as detailed in Table 4. The mediation analysis results showed that the 95% confidence intervals can significantly correlate with and partially explain the role of involution on depression–anxiety–stress and the mediating effect of competitive psychology did not include 0, indicating that involution in the study subjects can not only significantly correlate with and partially explain depression–anxiety–stress but can also significantly correlate with and partially explain depression–anxiety–stress through the mediating role of competitive psychology. The direct association of involution on depression–anxiety–stress was 0.430, and the mediating effect of competitive psychology (r = 0.798 × 0.137 = 0.110) accounted for 20.37% (0.110/0.540) of the total effect of 0.540. Effect size was interpreted according to conventional social science criteria: small (0.10), medium (0.30), and large (0.50). The mediating effect proportion of 20.37% represented a small-to-medium practical effect. The decomposition of the mediating effect is shown in Table 5, and the mediating effect model is illustrated in Figure 2.

## 4. Discussion

This study reveals that college student involution is significantly and positively associated with depression–anxiety–stress, and competitive psychology plays a mediating role in their correlational linkage. Our research findings indicate that college student involution is positively associated with depression–anxiety–stress, supporting Hypothesis H1. Previous studies have reported that students trapped in endless peer comparison alongside involution tend to experience elevated social anxiety and psychological imbalance (K. Li, 2024). Their research also noted that the state of involution coexists with social anxiety among college students. Involution is defined by excessive competition, heightened crisis awareness and persistent self-exhaustion. Under the combined influences of insufficient self-awareness, inaccurate self-evaluation and the gap between ideal and reality, this phenomenon is closely linked to poor mental health among young college students. Our results are consistent with the findings of Ma and Cheng (2023). Their qualitative research suggested that college students’ involution-related psychology correlates with peer competition, dormitory atmosphere and overall social environment. While involution may accompany improved academic performance for some students, it is also linked to a range of negative emotions including anxiety and depression. Collectively, these studies further confirm the close correlation between college students’ involution and their mental health status. The above findings offer empirical references for developing targeted mental health support strategies and optimizing campus environments for college students.

In addition, a study by Zheng et al. (2022) and Rabby et al. (2023) explored the relationship between anxiety and involution behaviors among college students in the post-pandemic era. Academic involution pressure stands as one factor that correlates with college students’ mental health. Consistent with Barbayannis et al. (2022), this study also identified a significant negative correlation between involution-related pressure and students’ mental health. The research results show that there is a significant positive correlation between college student involution and competitive psychology, supporting H2. In the current context, involution has become a typical manifestation of social anxiety among young people. It refers to involuntary group competition characterized by excessive participation, a strong sense of crisis and constant self-exhaustion. A cross-sectional study by Di Stasio et al. (2016) found that increased social comparison and competition are associated with student bullying and victimization, which also shows relevant links with classroom atmosphere. Long-term negative interpersonal experiences tend to co-occur with persistent psychological distress among students. These results align with the mixed-methods and two-stage Delphi study conducted by Thompson et al. (2022). Based on responses from 236 educators across primary, secondary and further education, the research indicated that competition and social comparison are correlated with students’ psychological distress. Thompson et al. (2022) also proposed that institutional pressures, curriculum adjustments and staffing changes may relate to intensified student competition and the prevalence of involution.

The research results show that there is a positive and significant correlation between college student competitive psychology and depression–anxiety–stress, supporting H3. This observed correlation may be explained by individual psychological regulation traits, which are linked to competitive tendencies and further connected to anxiety, depression and stress symptoms. Our findings are supported by Mu et al. (2019), who surveyed 825 undergraduates in Anhui Province and found a high prevalence of anxiety and depression among college students. Their work suggested that inadequate psychological regulation correlates with atypical competitive tendencies and subsequent negative emotional experiences. Their work provides references for universities to attach importance to students’ mental well-being and design corresponding supportive services.

Similarly, a survey of 360 medical undergraduates in Bangladesh (Islam et al., 2024) found that medical students tend to report more anxiety, depression and stress symptoms compared with peers in other majors. A systematic review and meta-analysis by W. Li et al. (2022) drew similar conclusions, and these results provide references for formulating mental health prevention and support plans for college students.

The research results show that competitive psychology plays a mediating role between involution and anxiety–depression–stress, supporting H4. The results indicate that college student involution relates to mental health problems such as anxiety, depression and stress via its association with competitive psychology. Involution presents mixed correlates for college students. It creates a fiercely competitive campus environment where students compete for limited educational resources including learning opportunities and postgraduate admission quotas. To pursue better grades and rankings, some students engage in excessive study and sacrifice rest time. Dissatisfaction with academic performance often coexists with inferiority and depressive feelings. The pervasive involution atmosphere is associated with widespread anxiety and stress, as most students worry about falling behind their peers. Involution, marked by excessive competition, crisis awareness and self-exhaustion, shares close correlations with social anxiety, and the two phenomena often appear simultaneously. This view is supported by Y. S. Wang et al. (2025), who reported that peer competition is an important correlational factor linked to college students’ involution. Continuous social comparison among classmates is associated with the emergence of involution tendencies. Against the backdrop of rapid social development, involution has become prevalent among contemporary college students. Many students are influenced by social trends and fall into passive competition for academic performance and job opportunities, and this group tendency correlates with the formation of collective involution-related cognition (Tian, 2025; Rong et al., 2023).

At the methodological level, this study employed a large-sample cross-sectional survey. By integrating social comparison theory and conservation of resources theory, we constructed a theoretical model to describe the correlational relationships between the key variables. We further clarified the mediating role of competitive psychology, distinguishing behavioral competition and cognitive–emotional competitive psychology, as well as their different correlational links with mental health. This distinction refines the conceptual framework for involution research. Additionally, this mediation model was verified among Chinese college students, which expands the research scope of involution across different cultural contexts. Involution in China carries unique cultural characteristics, such as collectivist peer comparison and family expectations under the one-child policy. This study provides empirical evidence to illustrate how these cultural factors correlate with college students’ mental health.

As a cross-sectional study, this research cannot establish definite causal relationships. Nevertheless, rigorous mediation analysis and effect size decomposition offer preliminary correlational evidence and effect size references for future longitudinal studies and relevant practical exploration. We fully acknowledge the limitations of cross-sectional mediation design and have elaborated the interpretation scope of all results and future research directions in the Section 4.3.

### 4.1. Recommendations for Future Research

Future research could consider employing a longitudinal research design to track changes in involution, competitive psychology, and mental health among college students over different time periods, in order to better understand the dynamic relationships between these variables. It should also expand the sample scope to include more regions and types of higher education institutions, thereby enhancing the generalizability of the research findings. Intervention-related research may be conducted in follow-up work to explore potential programs relevant to involution and competitive psychology. For example, researchers could design programs aimed at reducing competitive pressure and enhancing psychological resilience, and explore their potential relevance to students’ mental states. Qualitative methods, such as in-depth interviews or focus group discussions, should be incorporated to gain a deeper understanding of the psychological experiences and coping strategies of college students in the context of involution and competition. Further exploration of the correlational mechanisms linking involution and competitive psychology to college students’ mental health should be undertaken, considering other potential mediating or moderating variables, such as social support and self-efficacy.

### 4.2. Clinical Implications for Education Managers

Education managers may refer to the present findings to create a supportive and inclusive learning environment that reduces excessive competition. This can be achieved by encouraging cooperative learning and providing mental health support services. They could also take these results as a reference to optimize mental health education, helping students identify and manage stress associated with involution and competition, and offering training and counseling services focused on stress management and emotional regulation. The education evaluation system could be re-examined and adjusted to avoid an overemphasis on grades and rankings. Students could be encouraged to develop in a well-rounded manner, reducing competitive pressure driven by a single evaluation criterion. It is suggested that educators improve their ability to recognize students’ mental health concerns and provide supportive guidance, so as to better assist students in daily study and life. Communication and cooperation with parents could be strengthened to jointly pay attention to students’ psychological well-being and relieve excessive family pressure.

### 4.3. Limitations of the Study

It should be emphasized that this study adopts a cross-sectional design, which cannot establish temporal sequence or causal relationships. The present results only demonstrate that the observed data are statistically consistent with the hypothesized mediation model. No definitive causal interpretation should be made, and we do not claim that involution is associated with competitive psychology or psychological distress. Reciprocal or reverse associations among these variables are still possible. This study adopted a cross-sectional design, which precludes the determination of causal relationships between involution, competitive psychology, and mental health. Future research should employ a longitudinal design to verify these relationships. This study adopted convenience sampling and only recruited participants from three universities in Anhui Province. The sample is regionally limited and cannot fully represent all college students across China. Therefore, the research findings should be cautiously generalized to other regions and different types of universities. Future research could expand the sampling scope to cover multiple provinces and different levels of universities to improve the generalizability of the conclusions. The data in this study were based on self-reports from college students, which may be subject to certain subjective biases. Future research could incorporate multiple data sources, such as teacher evaluations and academic performance, to improve the reliability of the results. Although this study found that competitive psychology plays a mediating role between involution and mental health, there may be other unidentified mediating variables. Future research should further explore these potential mediating mechanisms. This study examined only the mediating role of competitive psychology, yet the mechanisms through which involution affects mental health may be multifaceted. According to self-determination theory, the frustration of autonomy, competence, and relatedness needs may all serve as important mediators; according to transactional theory of stress, cognitive appraisal and coping strategies may also play mediating roles. Although competitive psychology is theoretically a direct mediating mechanism, the existence of other parallel pathways cannot be ruled out. Future research should construct more complex multiple mediation models to compare the relative contributions of different mechanisms. In addition, this study only adopted Harman’s single-factor test for common method bias detection, without adopting more rigorous methods such as common latent factor or CFA-based comparison. Future studies could employ multiple statistical approaches to further control common method bias. Moreover, this study lacked CFA, AVE and HTMT analyses for discriminant validity assessment. Follow-up studies should supplement these tests to improve the robustness of relevant conclusions.

## 5. Conclusions

This study explored statistical associations among college student involution, competitive psychology, and anxiety–depression–stress, as well as the mediating pattern based on cross-sectional data from three universities in Anhui Province. The results show that all variables were significantly correlated, and the data fit the hypothesized mediating model of competitive psychology between involution and anxiety–depression–stress. Given the cross-sectional design, definitive causal pathways cannot be established, and competitive psychology should only be interpreted as a correlational mediational link rather than a deterministic influencing factor. The present findings provide theoretical references for mental health management in local Anhui universities, rather than generalized implications for national educational policy. Relevant practical reflections are derived from statistical associations and lack empirical verification of intervention effects. Future longitudinal and exploratory intervention research is needed to further verify potential correlational mechanisms and develop targeted mental health promotion approaches.

## Figures and Tables

**Table 1:** Full demographic characteristics of participants (*N* = 592).

Variable	Category	Total (*n* = 592)
Gender, *n* (%)	Male	283 (47.80)
	Female	309 (52.20)
Ethnicity, *n* (%)	Han	509 (85.98)
	Minorities	83 (14.02)
Only Child, *n* (%)	No	398 (67.23)
	Yes	194 (32.77)
Serving as Class or Student Council Officer, *n* (%)	No	368 (62.16)
	Yes	224 (37.84)
Family Atmosphere, *n* (%)	Harmonious	339 (57.26)
	Average or Poor	253 (42.74)
Romantic Status, *n* (%)	In a Relationship	214 (36.15)
	Single	378 (63.85)
Interpersonal Relationships, *n* (%)	Good	287 (48.48)
	Average or Poor	305 (51.52)
Age, mean (SD)	/	20.94 (2.43)
Average Daily Extracurricular Study Time in the Past Week, mean (SD)	/	2.02 (0.97)
Average Weekly Frequency of Exercise, mean (SD)	/	2.81 (1.14)
Daily Mobile Phone Usage Time, mean (SD)	/	2.69 (0.89)
Competitive Psychology Total Score, mean (SD)	/	65.60 (19.65)

**Table 2:** Scores of involution, competitive psychology, and depression–anxiety–stress in the study subjects.

Variables	Mean ± SD (*n* = 592)
Total Involution Scale Score, Mean ± SD	73.98 ± 26.13
Achievement Motivation Involvement Dimension, Mean ± SD	23.16 ± 7.99
Reward-Oriented Involvement Dimension, Mean ± SD	14.34 ± 5.27
Passive Involvement Dimension, Mean ± SD	36.48 ± 13.79
Total Competitive Psychology Scale Score, Mean ± SD	65.60 ± 19.67
Excessive Competitive Attitude Dimension, Mean ± SD	20.48 ± 7.11
Competitive Motivation Dimension, Mean ± SD	18.70 ± 6.20
Competitive Interpersonal Relationships Dimension, Mean ± SD	11.47 ± 3.88
Personal Development Competitive Attitude Dimension, Mean ± SD	14.96 ± 5.26
Total Depression–Anxiety–Stress Scale Score, Mean ± SD	44.78 ± 18.34
Stress Dimension, Mean ± SD	15.16 ± 6.21
Anxiety Dimension, Mean ± SD	14.84 ± 6.25
Depression Dimension, Mean ± SD	14.78 ± 6.39

SD: standard deviation.

**Table 3:** Correlation analysis of involution, competitive psychology, and depression–anxiety–stress in the study subjects.

Variable	Total Involution Score	Total Competitive Psychology Score	Total Depression–Anxiety–Stress Score
Total Involution Score	1.000		
Total Competitive Psychology Score	0.827 ***	1.000	
Total Depression–Anxiety–Stress Score	0.638 ***	0.580 ***	1.000

Note: *** represents significance levels of *p* < 0.001.

**Table 4:** Regression analysis results of involution and competitive psychology on depression–anxiety–stress.

Variables	Depression–Anxiety–Stress (Dependent Variable)			Competitive Psychology (Dependent Variable)			Depression–Anxiety–Stress (Dependent Variable)		
	β	t	*p*	β	t	*p*	β	t	*p*
Constant	-	−3.891	0.000	-	2.278	0.023	-	−4.154	0.000
Gender (Female vs. Male)	−0.099	−3.476	0.001	0.001	0.035	0.972	−0.099	−3.500	0.001
Age	0.096	3.256	0.001	−0.047	−1.946	0.052	0.102	3.485	0.001
Ethnicity (Minority vs. Han)	0.086	2.982	0.003	−0.050	−2.092	0.037	0.092	3.225	0.001
Only Child (No vs. Yes)	0.065	2.209	0.028	0.049	2.021	0.044	0.058	1.984	0.048
Class or Student Union Officer (No vs. Yes)	−0.005	−0.170	0.865	0.026	1.110	0.267	−0.008	−0.297	0.766
Family Atmosphere (Average or Poor vs. Harmonious)	0.212	7.140	0.000	0.047	1.905	0.057	0.206	6.941	0.000
Romantic Relationship Status (Single vs. In a Relationship)	−0.097	−3.366	0.001	−0.033	−1.381	0.168	−0.092	−3.222	0.001
Interpersonal Relationships (Average or Poor vs. Good)	0.147	4.971	0.000	0.033	1.369	0.171	0.142	4.835	0.000
Average Daily Extracurricular Study Time in the Past Week	−0.031	−1.064	0.288	−0.041	−1.715	0.087	−0.025	−0.872	0.384
Average Weekly Exercise Frequency	−0.102	−3.591	0.000	−0.023	−0.971	0.332	−0.099	−3.497	0.001
Average Daily Mobile Phone Usage Time	0.031	1.093	0.275	−0.041	−1.744	0.082	0.036	1.294	0.196
Total Involution Score	0.540	18.054	0.000	0.798	32.240	0.000	0.430	8.658	0.000
Total Competitive Psychology Score							0.137	2.747	0.006
R^2^	0.56			0.699			0.565		
Adjusted R^2^	0.551			0.692			0.556		
F Value	F = 61.337, *p* = 0.000			F = 111.829, *p* = 0.000			F = 57.839, *p* = 0.000		

Note: All 95% bias-corrected and accelerated (BCa) confidence intervals for the mediation model were generated via bootstrap analysis (5000 resamples). Detailed results for total, direct and indirect effects are presented in Table 5 (Mediation Summary Table).

**Table 5:** Decomposition of the total effect of involution on depression–anxiety–stress, direct effect, and mediating effect of competitive psychology.

Item	Effect Size	Standard Error	Lower Limit	Upper Limit	Proportion of Effect
Total Effect	0.540	0.030	0.481	0.598	100.00%
Indirect Effect	0.110	0.044	0.028	0.198	20.37%
Direct Effect	0.430	0.050	0.333	0.528	79.63%

Note: All effect sizes presented are standardized.

## Data Availability

The datasets generated and/or analyzed during the current study are not publicly available due to multiple datasets being required for other related research. But are available from the corresponding author on reasonable request.
